# Case report: Birth achieved after effective ovarian stimulation combined with dexamethasone in a patient with resistant ovary syndrome

**DOI:** 10.1186/s13048-022-00976-4

**Published:** 2022-04-07

**Authors:** Huiying Li, Tianli Chang, Hongbei Mu, Wenpei Xiang

**Affiliations:** 1 Institute of Reproductive Health, Tongji Medical College, Huazhong University of Science and Technology, Wuhan, Hubei China; 2 Center of Reproductive Medicine, Tongji Medical College, Huazhong University of Science and Technology, Wuhan, Hubei China

**Keywords:** Resistant ovary syndrome, Infertility, Immunosuppressant, Case report, Dexamethasone

## Abstract

**Background:**

Resistant ovary syndrome (ROS) is a rare endocrine disorder and there have been few reports of live births by affected patients. As gonadotropin resistance leads immature oocytes, some researchers reported few live births with in vitro maturation (IVM) of oocytes, but IVM is not always successful in ROS patients. Here, we report an original case of ROS, associated with Ig-FSHR in the serum, who achieved a live birth following ovarian stimulation combined with dexamethasone treatment.

**Case presentation:**

The 30-year-old woman presented with secondary amenorrhea and infertility. Her serum FSH levels were found to be higher than normal, but in discordance with a normal anti-Müllerian hormone (AMH) level and antral follicle count. Genetic investigation found no mutations potentially affecting FSHR. With reference of previous ROS studies, the patient’s serum was analyzed for antibodies directed against FSHR and dot blot analysis showed strong reactivity with FSHR. Then, dexamethasone was proposed to the patient, and she successfully became pregnant, finally delivering a healthy girl by caesarean section.

**Conclusion:**

To our best knowledge, this is the first report of the successful treatment of ROS using ovarian stimulation combined with dexamethasone. In some cases of ROS, high doses of exogenous gonadotropins in combination with immunosuppressive therapy could be an effective approach.

## Background

Resistant ovary syndrome (ROS), also mentioned as “Gonadotropin-Resistant Ovary” syndrome [1], is a rare endocrine disorder that was first described by Moraes-Ruehsen and Seegar Jones in 1967 [2]. They found that some cases of premature ovarian failure (POF) had milder symptoms and were not sensitive to gonadotropin. They referred to this condition as resistant ovary syndrome [3],, which is characterized by amenorrhea along with normal sexual characteristics and follicle number. Notably, the ovaries of ROS patients are not sensitive to high doses of exogenous gonadotropin. Today, a normal AMH value is also used as a diagnostic criterion for ROS [4] and is considered the main characteristic that distinguishes it from POF [5].

Although ROS is diagnostically well defined, slow progress has been made in its treatment. At present, pregnancies obtained with the patient’s own oocytes are mainly achieved through hormone therapy [6–12] or in vitro fertilization (IVF) after ovulation stimulation [4], as well as the acquisition of valid embryos through in vitro maturation (IVM) [13–15]. However, patients suffering from ROS who fail to produce oocytes following treatment have to be put on a waiting list for oocyte donation [16–22]. Here, we present the case of a patient diagnosed with ROS, who successfully became pregnant and delivered a healthy baby after treatment with high-dose gonadotropin and dexamethasone.

## Case presentation

**Fig. 1:**
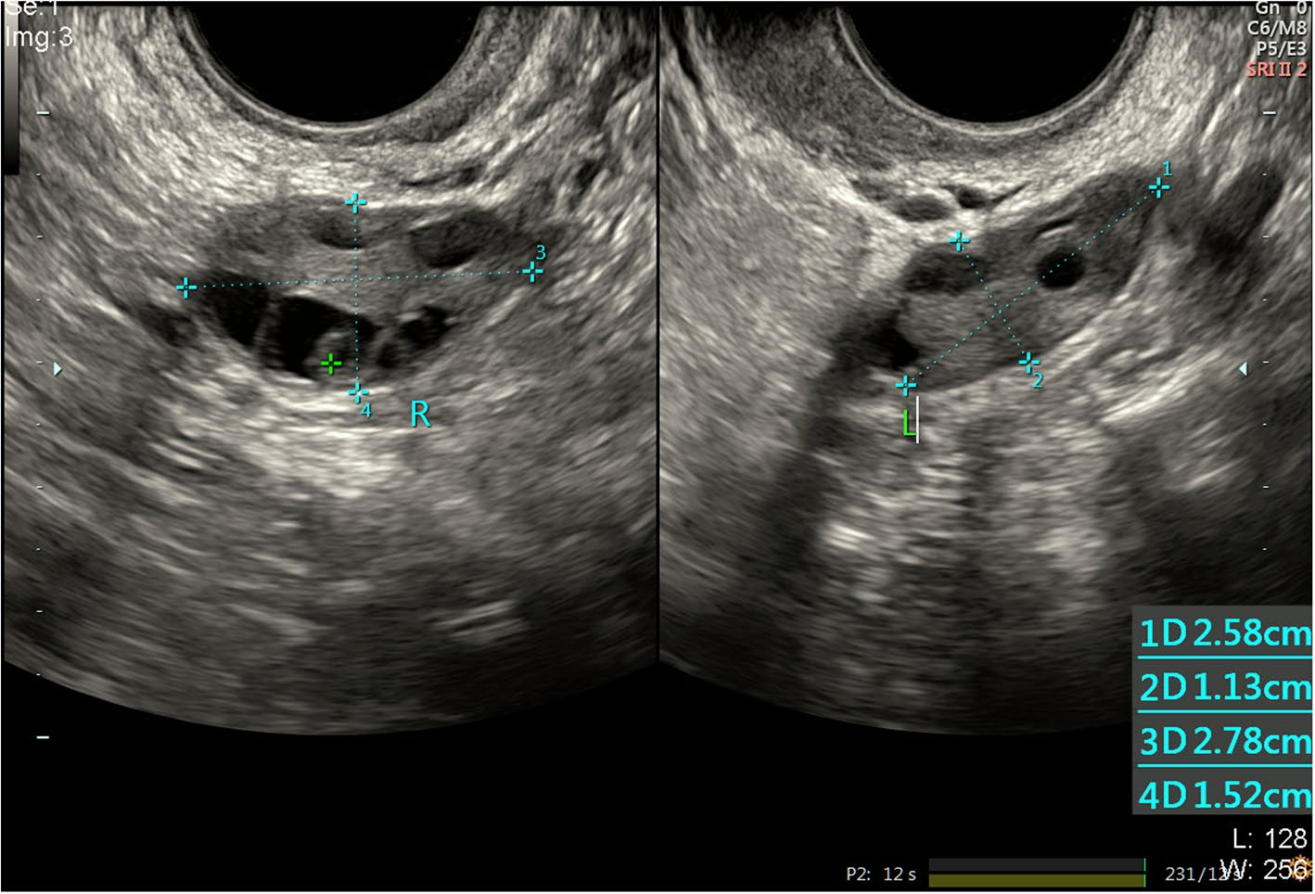
Transvaginal ultrasound scans of the bilateral ovaries. The size of the left and right ovaries was 2.7 × 1.6 cm and 3.2 × 1.4 cm, respectively. The number of antral follicles in the left and right ovary was 12 and 16, respectively, in line with the normal AMH level but in contrast with the high serum FSH and LH levels

A 30-year-old woman with a body mass index (BMI) of 22.5 was hospitalized due to secondary amenorrhea and infertility. Having been married for 10 years with regular intercourse, she delivered a baby girl in 2009. After that, the patient could not become pregnant again in spite of not using contraception. She experienced amenorrhea for 6 years after giving birth, and had been treated with drugs and contraceptive rings without success. The patient could only adjust her menstrual cycle with medication. The results of basic endocrine examination showed that the serum level of follicle-stimulating hormone (FSH) was high. Examination in April, 2019 (Table 1) indicated that serum FSH was above normal, while the anti-Müllerian hormone (AMH) concentration remained normal. Ultrasound scanning indicated that the uterine volume was relatively small (4.0 × 3.9 × 3.3 cm), while both ovaries were normal in size and more than 10 antral follicles were observed in both ovaries (Fig. 1). Blood tests and genetic analysis excluded lupus erythematosus, multiglandular insufficiency, diabetes, myasthenia gravis, and chromosomal abnormalities (Fragile X syndrome, Turner syndrome, and Swyer syndrome). The patient had a normal karyotype of 46, XX. Sanger sequencing did not identify associated candidate variants in the FSHR gene. Serological tests, combined with clinical diagnosis and the characteristics of the patient’s infertility suggested ROS.

**Table 1:** Laboratory test for hormonal profile

	2019-3-14	2019-4-12	Normal ranges
FSH	42.37	40.8	Early follicular phase (2.5–10.2) IU/L
LH	15.13	11.49	Early follicular phase (1.9–12.5) IU/L
E2	15	11	Early follicular phase (11–69) pg/mL
AMH	6,29	6.14	(2.1–6.5) ng/mL
T	1.8	NA	(0.7–3.1) nmol/L
P	0.15	NA	Early follicular phase (0.38–2.28) ng/mL
PRL	408.94	NA	(72–511) mIU/L
FT3	4.92	NA	(4–10) pmol/L
FT4	18.39	NA	(9–25) pmol/L
TSH	3.01	NA	(0.3–5.0) mIU/L
AFC (2–12 mm)	> 20	28	(12–24)

*Abbreviations*: *FSH* follicle-stimulating hormone, *LH* luteinizing hormone, *E2* estradiol, *AMH* anti-müllerian hormone, *T* testosterone, *P* progesterone, *PRL* prolactin, *FT3* free triiodothyronine, *FT4* free thyroxine, *TSH* thyroid stimulating hormone, *AFC* antral follicle count, *NA* not applicable

According to the 5th semen analysis standard of the world health organization, the husband’s sperm concentration and motility were in the normal range, and sperm acrosomal enzyme activity was normal. The study was conducted in accordance with the ethical guidelines of the institution and with the informed consent of the patient.

**Fig. 2:**
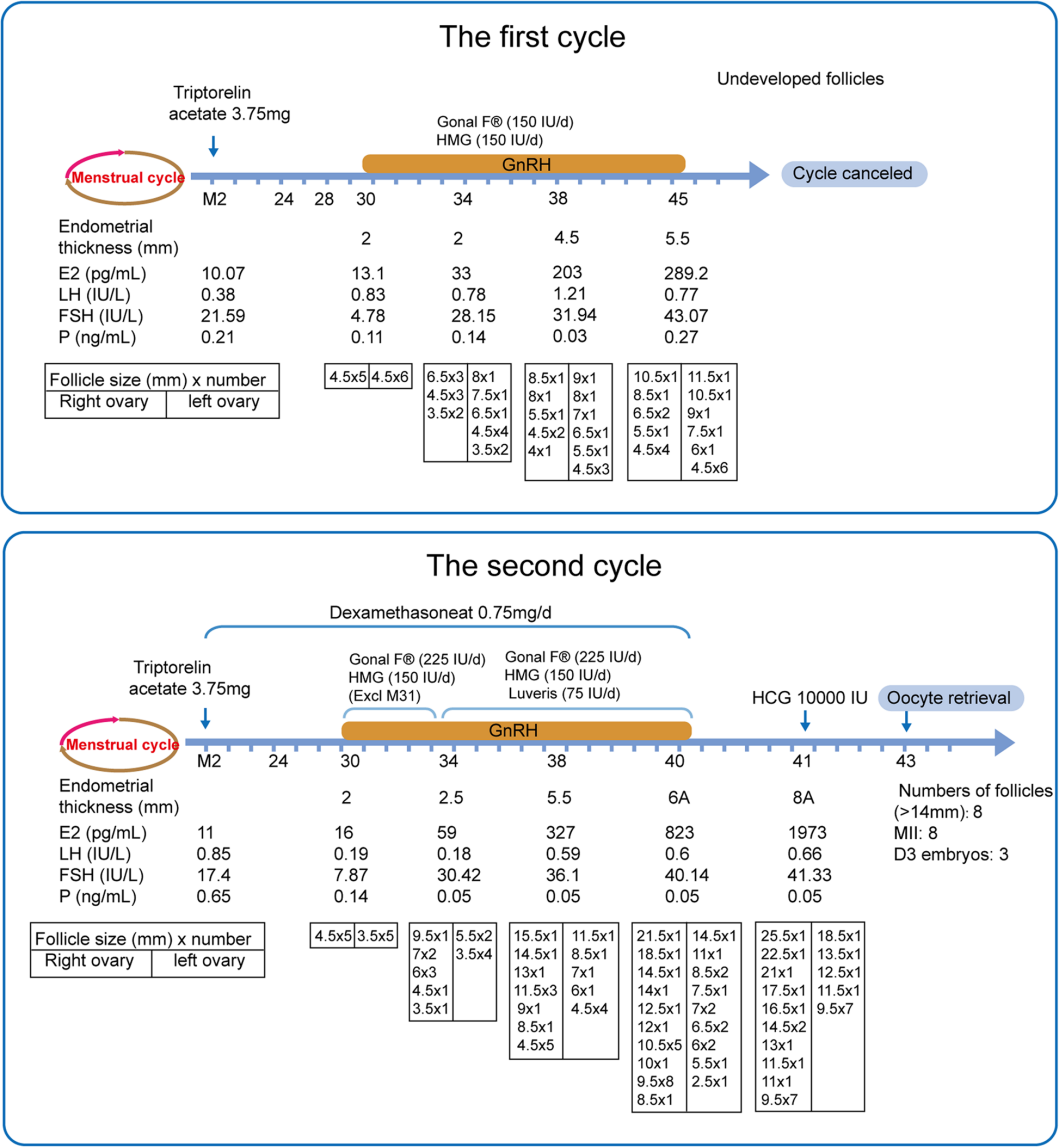
Diagram of two ovarian hyperstimulation cycles. GnRH, gonadotropin-releasing hormone; HCG, human chorionic gonadotropin; HMG, human menopausal gonadotropin; FSH, follicle-stimulating hormone; LH, luteinizing hormone; E2, estradiol; P, progesterone

After admission on March 4th, 2019, the patient underwent two cycles of ovarian hyperstimulation treatment (Table 2, Fig. 2). The first one (May 19, 2019) was initiated with 3.75 mg of GnRH analogue triptorelin acetate injection (Ferring, Switzerland), followed by gonadotropin (300 IU/d, 15d) on cycle day 30. During the ovarian hyperstimulation period, the follicle growth was followed by ultrasound scanning, and the serum hormone levels were determined at the same time. Unfortunately, after 15 days of stimulation, no follicles larger than 14 mm were seen, and this treatment cycle was cancelled.

**Table 2:** Cycle characteristics and results in patient with resistant ovary syndrome

Cycle No.	Protocol	Hormon	Total gonadotropin (IU)	Days of stimulation	Serum E2 on oocyte retrieval day (pg/mL)	Numbers of follicles (> 14 mm)	MII	D3 embryo	The Result
1	Long GnRH agonist	Triptorelin Acetate Injection	3000	15	NA	0	NA	NA	Cycle the cancel
2	Long GnRH agonist	Triptorelin Acetate Injection	4800	11	1973	8	8	3	Pregnant

*Abbreviations*: *GnRH* gonadotropin-releasing hormone, *MII* metaphase II oocytes, *D3* the third day, *IU* international unit, *NA* not applicable

**Fig. 3:**
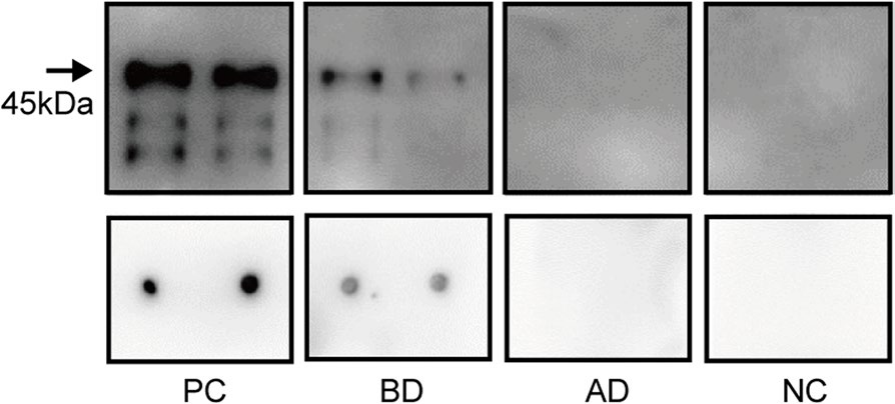
Immunoprecipitation followed by western blotting and dot blot analysis of anti-FSHR autoantibodies in the serum of the patient. A polyclonal antibody against FSHR (Abcam, ab113421) in TBST buffer (containing 3% BSA) was used as positive control serum with anti-FSHR autoantibodies, a healthy person’s serum was used as negative control, and sera from the patient before and after the dexamethasone treatment were used to immunoprecipitate recombinant FSHR protein purified from *E. coli* (ImmunoClone, IC8974-A). Samples comprising 0.6 μg of FSHR protein per well were separated on 10% SDS-PAGE gels and then transferred to nitrocellulose membranes. For the dot blot assay, FSHR protein (100 ng/dot) was applied onto nitrocellulose membranes. The membranes were incubated overnight at 4 °C with serum from the patient and the healthy control, followed by incubation with a horseradish peroxidase (HRP)-conjugated anti-human secondary antibody (Proteintech, SA00001–11) or anti-rabbit secondary antibody (Proteintech, SA00001–2). Stained bands or dots were visualized using Omni-ECL reagent (EpiZyme, SQ201). PC: positive control; BD: before the dexamethasone treatment; AD: after the dexamethasone treatment. NC: negative control; FSHR: follicle-stimulating hormone receptor

Due to the failure of the first cycle and a lack of FSHR associated variants in the genetic investigation, we tested the patient’s serum for antibodies directed against FSHR via dot blot analysis, which showed strong reactivity with FSHR (Fig. 3). Consequently, we adjusted the procedure for the second cycle. During the whole period of downregulation and controlled ovarian hyperstimulation, the patient was orally administered dexamethasone at 0.75 mg daily. The second cycle was started on July 6, 2019, at which time the patient was first given a 3.75 mg injection of triptorelin acetate for downregulation on the second day of menstruation. Controlled ovarian hyperstimulation was initiated on day 30 with daily subcutaneous injections of 375 IU of gonadotropin (Gonal F® 225 IU/d plus HMG 150 IU/d) for 3 days, which was then increased to 525 IU (Gonal F® 225 IU/d, HMG 225 IU/d and Luveris 75 IU/d) for 7 days. During the stimulation period, the patient underwent regular ultrasound follicle tracking and hormone measurements (estradiol, luteinizing hormone, follicle-stimulating hormone and progesterone) to monitor follicular maturation. A subcutaneous injection of hCG 10,000 IU (Livzon Pharmaceuticals, China) was administered and oocyte retrieval was scheduled 36 h later, ultrasound guided transvaginal follicular aspiration was performed under negative pressure of 110 mmHg (14.7 kPa) using a single lumen aspiration needle (Cook; William Cook Australia Pty Ltd., Australia). A total of 8 Metaphase II (MII) oocytes were collected. After in vitro fertilization, 3 embryos were vitrified and cryopreserved, and the remaining embryos were discarded.

After 2 months following the second ART cycle, a hormone replacement cycle for endometrial preparation was started on day 3 of menstrual cycle with estradiol valerate tablets (Bayer, Germany, 4 mg for 5 days and then 6 mg for the same period). The addition of oral estradiol (Bayer, Germany) at a dose of 8 mg daily for the next 3 days was successful in achieving an endometrial thickness of 9 mm. The serum E2 on day 16 was 346 pg/ml. Progesterone (0.05 ng/ml) and human chorionic gonadotropin (HCG, 10000 IU) were injected at night. Then, daily progesterone luteal support with vaginal tablets containing 40 mg of progesterone (Utrogestan, Besins, Paris, France) was started.

One embryo was thawed on day 20 (14 CII, grade II embryo with 14 cells) and transplanted. The serum value of β-hCG was 246.7 mIU/mL on the thirteenth day after the embryo transfer, and vaginal ultrasonography showed clinical pregnancy after 28 days. The pregnancy evolved without complications until the 35th week, at which point the patient exhibited oligohydramnios and gave birth to a baby girl by Caesarean section. The baby weighed 2200 g and was in good health.

## Discussion

Here, we report a successful pregnancy following treatment of a ROS patient. The first ovarian hyperstimulation cycle inducted little ovarian response. Prior to the second cycle, we detected the presence of Ig-FSHR in the serum of the patient. Then, we started the second cycle of ovarian hyperstimulation with the addition of the immunosuppressant dexamethasone to reduce the FSHR antibody levels in peripheral blood. The patient finally obtained 8 mature oocytes and 3 embryos were frozen. Two months later, one embryo was thawed and transferred, and finally the patient achieved clinical pregnancy and live birth.

Infertility is a pressing problem for people of reproductive age with ROS, and the chances of having children with their own oocytes are unpredictable. Different ROS treatments have been explored. In previous cases of successful pregnancy, the treatments were mainly based on hormone replacement therapy, controlled ovarian hyperstimulation, and IVM (Table 3). In early cases, different hormone replacement therapies were often used to restore normal menstruation (15–21). Later, it was found that patients with secondary ROS could achieve pregnancy through periodic estrogen administration [6]. For hormonal therapy of ROS, estradiol, clomiphene, and high-dose hMG are commonly used [18]. However, the response of ROS patients shows significant individual differences. Some cases of ROS could even recover spontaneously [9], while other ROS cases generally presented with amenorrhea, but could become pregnant through hormone therapy more than once [10]. Therefore, the treatment of ROS is also dependent on the severity of the disease.

**Table 3:** Cases of ROS received pregnancy through different treatments

Author	Patient No.	Age at intake	Type of infertility	BMI (kg/m2)	Basal AFC	AMH (μg/L)	Ovarian histology	E2	FSH	Ig-FSHR	Infertility treatments before pregnant	Medication/hormonal pretreatment	Result
Amos, W. L., Jr. (1985) [12]	1	41	Secondary	NA	NA	NA	NA	NA	88.4 IU/L	NA	HRT	Estrogens, medroxyprogesterone acetate	Liveborn
Jequier, A. M. (1990) [6]	2	28	Secondary^a^	NA	NA	NA	NA	31–52 pmol/L	125 U/L	NA	HRT	4 cycles (Mestranol + Norethisterone)	Liveborn
	3	30	Secondary^a^	NA	NA	NA	normal ovarian stroma and follicles	76 pmol/L	range seen in postmenopausal women	NA	HRT	Mestranol + Norethisterone	Normal pregnancy
Nawroth, F. and R. Sudik (1999) [8]	4	32	Secondary	NA	NA	NA	NA	NA	NA	NA	HRT	2 mg estradiol valerate and 2 mg estradiol valerate/0.15 mg levonorgestrel	Liveborn
Mueller, A., et al. (2003) [9]	5	26	Primary	NA	NA	NA	normal density of follicles	NA	70 IU/L	NA	HRT	2 mg estradiol valerate and 0.5 mg norgestrel per day administered sequentially	Liveborn
Aslam, M. F., et al. (2004) [10]	6	19	Secondary	NA	NA	NA	NA	NA	133.9 U/L	NA	HRT	2 mg estradiol valerate and 0.5 mg norgestrel	Twice liveborn
	7	24	Secondary	27	NA	NA	NA	NA	Higher than normal	NA	HRT	estradiol valerate and norgestrel	Normal pregnancy
Zielinska, D. and I. Rzepka-Gorska (2011) [11]	8	31	Secondary	NA	NA	NA	NA	18.1 pg/ml	58.2 IU/mL	NA	HRT	(spontaneous recovery of ovarian function after HRT)	liveborn
Ezeh, U. I. O. and A. J. Breeson (1995) [7]	9	32	22	NA	NA	NA	NA	39 pmol/L	95–115 IU/L	NA	Ovarian Hyperstimulation	eight ampoules of menotrophin (Pergonal) daily for 14 days	Liveborn
Rogenhofer, N., et al. (2015) [4]	10	26	Secondary	22	15	2.1	NA	28.7 pg/mL	50.8 U/mL	antibodies directed to hMG but not to recFSH	Controlled Ovarian Hyperstimulation and IVF	GnRH analogue Narfarelin, recombinant follitropin beta, hMG	liveborn
Grynberg, M., et al. (2013) [13]	11	29	primary	normal	23 and 18	4.50 and 4.36	NA	< 15	40.3 and 38.4 mIU/mL	NA	IVM	17ß-E2, hCG	liveborn
Li, Y., et al. (2016) [14]	12	33	Secondary^a^	NA	25	12.27	NA	260.57 pmol/l	41.99 IU/L	NA	IVM	estradiol valerate, hCG	liveborn
Galvao, A., et al. (2018) [15]	13	29	primary	27.7	37	8.6	NA	NA	27.7 IU/L	NA	IVM	none	liveborn
	14	36	primary	18.9	40	2.11	NA	NA	7.9 IU/L	NA	IVM	HP-hMG 150 IU/day for 5 days, hCG	liveborn
	15	23	primary	24.8	50	2.88	NA	NA	49.1 IU/L	NA	IVM	17ß-E2, hCG	Twice liveborn
C. Flageole., et al. (2019) [23]	16	31	primary	NA	19	3.24 ng/mL	NA	NA	43–62 IU/L	NA	IVM	hMG (Menopur®) plus rFSH (Gonal F®), hCG	liveborn

*Abbreviations*: *BMI* body mass index, *AFC* antral follicle count, *AMH* anti-müllerian hormone, *HRT* hormone replacement therapy, *IVM* in vitro maturation, *ART* assisted reproductive technologies, *rFSH* recombinant follicle stimulating hormone, *FSHR* follicle-stimulating hormone receptor, *GnRH* gonadotropin-releasing hormone, *hCG* human chorionic gonadotropin, *hMG* human menopausal gonadotropin, *HP-hMG* highly purified human menopausal gonadotropin, *17ß-E2* estradiol-17ß, *NA* not applicable

^a^One live birth after spontaneous birth

Some researchers considered that the different basic FSH levels can better reflect the degrees of ovarian follicle resistance to FSH than the follicle numbers [24]. In our opinion, although the grading of ROS according to FSH levels can preliminarily determine the severity of ROS, it is not rigorous, because there were only 6 cases in the report that put this idea forward. Moreover, the case reported by Galvao (14) also proved that a patient with normal FSH levels could show obvious resistance to gonadotropin stimulation. We therefore considered that the FSH level can only reflect the follicular reactivity to ovulation-inducing drugs from one side, which can only be used for reference in the selection of a treatment plan.

The etiology of ROS has not been elucidated, and its pathogenesis may be related to genetic or immunological factors. A number of studies have shown that FSHR mutations are associated with ROS [25–27]. For instance, patients with p.N680S mutation needed a higher dose of FSH stimulation to get the normal serum estrogen level [28–31], suggesting that FSHR (p.N680S) affects the sensitivity of ovaries to FSH, leading to partial “resistance” to FSH. In addition to genetic factors, immunological factors may also contribute to ROS. Studies have shown that there may be auto-antibodies against FSHR, which block the ovaries from responding to gonadotropin stimulation [32–37].

In fact, there was heterogeneity among different ROS patient, and the etiology may involve gene mutations [26, 27] and autoimmune disorders [36, 37]. ROS can only be treated effectively if the causes are clearly identified. Although it was reported that IVM may be a viable treatment option for ROS [15], the etiology of the patients was still unknown. The association between ROS and autoimmune disorders was first proposed in 1982 [36, 37]. The authors found that gonadotropin resistance has the same immune mechanism as myasthenia gravis, and they confirmed that the patient serum contained a substance similar to gamma globulin, which inhibited the specific binding of FSH to the receptor in vitro, possibly explaining the ovary’s non-response to gonadotropin stimulation. In 2004, Chiauzzi et al. found that all the ROS patients had circulating immune complexes in the serum, which might block the binding of FSH to its receptor [33].

Although there is evidence that ROS is linked to autoimmune disorders, few cases have been reported in which Ig-FSHR antibodies were detected or corresponding immunotherapy was administered to patients with ROS. In 2015, Rogenhofer described a ROS patient who achieved pregnancy by controlled stimulated ovulation. In their study, resistance to HMG signals was detected in the serum, so they chose recombinant follicle element beta and high purity HMG for ovarian stimulation and obtained a positive response, providing an illustrative case for symptomatic treatment on the basis of a defined cause [4].

In this study, we detected Ig-FSHR antibodies in the patient’s serum [4]. Using a standard long GnRH agonist scheme, with a large dose of gonadotropin combined with immunosuppressive dexamethasone, the patient eventually achieved pregnancy. This result is similar to the treatment method reported by Riestenberg et al. in a primary ovarian insufficiency (POI) patient with polyglandular autoimmune syndrome (PGAS) type 2 [38]. They report a case of successful COH and oocyte cryopreservation with the use of short-term, high-dose prednisone for temporary immune sup-pression in a patient with presumptive Ig-FSHR associated POI who was initially resistant to stimulation with maximal dosing of gonadotropins, which means the use of corticosteroids for suppression of aberrant autoimmune antibodies may be one of the treatments. Our case report may provide a valuable reference for further ROS treatment.

In general, ROS is closely related to autoimmunity, so we should identify the cause and then carry out targeted treatment, which may greatly increase the success rate of pregnancy in ROS patients. For some patients with abnormal immunity causing ROS, treatment with a large dose of gonadotropin in combination with immunosuppressive agents may be successful.

## Data Availability

Full availability of data and material are declared. Extra data is available by emailing Wenpei Xiang.

## Abbreviations

ROS: Resistant ovary syndrome
IVF: In vitro fertilization
POF: Premature ovarian failure
FSHR: Follicle-stimulating hormone receptor
FSH: Follicle-stimulating hormone
LH: Luteinizing hormone
E2: Estradiol
AMH: Anti-müllerian hormone
T: Testosterone
P: Progesterone
PRL: Prolactin
FT3: Free triiodothyronine
FT4: Free thyroxine
TSH: Thyroid stimulating hormone
AFC: Antral follicle count
IU: International unit
GnRH: Gonadotropin-releasing hormone
MII: Metaphase II oocytes
D3: The third day
14 CII: Grade II embryo with 14 cells
BMI: Body mass index
HRT: Hormone replacement therapy
IVM: In vitro maturation
ART: Assisted reproduction technology
rFSH: Recombinant follicle-stimulating hormone
FSHR: Follicle-stimulating hormone receptor
GnRH: Gonadotropin-releasing hormone
hCG: Human chorionic gonadotropin
hMG: Human menopausal gonadotropin
HP-hMG: Highly purified human menopausal gonadotropin
17ß-E2: Estradiol-17ß
NA: Not applicable

## References

[1] Evers JL, Rolland R (1981). The gonadotrophin resistant ovary syndrome: a curable disease?. Clin Endocrinol.

[2] de Moraes-Ruehsen M, Jones GS (1967). Premature ovarian failure. Fertil Steril.

[3] Jones GS, De Moraes-Ruehsen M (1969). A new syndrome of amenorrhae in association with hypergonadotropism and apparently normal ovarian follicular apparatus. Am J Obstet Gynecol.

[4] Rogenhofer N (2015). Effective ovarian stimulation in a patient with resistant ovary syndrome and Antigonadotrophin antibodies. Am J Reprod Immunol.

[5] Kallio S (2012). Anti-Mullerian hormone as a predictor of follicular reserve in ovarian insufficiency: special emphasis on FSH-resistant ovaries. Hum Reprod.

[6] Jequier AM (1990). Conception in the resistant ovary syndrome occurring during hormone replacement therapy: a report of 2 cases. Aust N Z J Obstet Gynaecol.

[7] Ezeh UIO, Breeson AJ (1995). Spontaneous pregnancy following pretreatment with gonadotrophins in a patient with resistant ovary syndrome. J Obstetr Gynaecol.

[8] Nawroth F, Sudik R (1999). Pregnancy and hormone replacement therapy in a patient with resistant ovary syndrome. Zentralbl Gynakol.

[9] Mueller A (2003). Spontaneous normalization of ovarian function and pregnancy in a patient with resistant ovary syndrome. Eur J Obstet Gynecol Reprod Biol.

[10] Aslam MF, Gilmour K, McCune GS (2004). Spontaneous pregnancies in patients with resistant ovary syndrome while on HRT. J Obstetr Gynaecol.

[11] Zielinska D, Rzepka-Gorska I (2011). Pregnancy in a patient with resistant ovary syndrome - a case report. Ginekol Pol.

[12] Amos WL (1985). Pregnancy in a patient with gonadotropin-resistant ovary syndrome. Am J Obstet Gynecol.

[13] Grynberg M (2013). First birth achieved after in vitro maturation of oocytes from a woman endowed with multiple Antral follicles unresponsive to follicle-stimulating hormone. J Clin Endocrinol Metab.

[14] Li Y (2016). Successful live birth in a woman with resistant ovary syndrome following in vitro maturation of oocytes. J Ovarian Res..

[15] Galvao A (2018). In vitro maturation (IVM) of oocytes in patients with resistant ovary syndrome and in patients with repeated deficient oocyte maturation. J Assist Reprod Genet..

[16] Koninckx PR, Brosens IA (1977). The “gonadotropin-resistant ovary” syndrome as a cause of secondary amenorrhea and infertility. Fertil Steril.

[17] Starup J, Pedersen H (1978). Hormonal and ultrastructural observations in a case of resistant ovary syndrome. Acta Endocrinol.

[18] Mori H (1985). Sporadic ovulation in a case of resistant ovary syndrome. Asia-Oceania J Obstetr Gynaecol.

[19] Fraser IS (1986). Resistant ovary syndrome and premature ovarian failure in young women with galactosaemia. Clin Reprod Fertil.

[20] Twigg S, Wallman L, McElduff A (1996). The resistant ovary syndrome in a patient with galactosemia: a clue to the natural history of ovarian failure. J Clin Endocrinol Metab.

[21] Sung M (2012). A case of resistant ovary syndrome. Obstetr Gynecol Sci.

[22] Tolino A, Romano L, Montemagno U (1984). Resistant ovary syndrome and fertility. Acta Eur Fertil.

[23] Flageole C (2019). Successful in vitro maturation of oocytes in a woman with gonadotropin-resistant ovary syndrome associated with a novel combination of FSH receptor gene variants: a case report. J Assist Reprod Genet.

[24] Huang B (2017). Grading and etiology of ovarian resistant syndrome. Chin J Pract Gynecol Obstetr.

[25] Aittomaki K (1995). Mutation in the follicle-stimulating-hormone receptor gene causes hereditary hypergonadotropic ovarian failure. Cell.

[26] Latronico AC, Arnhold IJP (2006). Inactivating mutations of LH and FSH receptors--from genotype to phenotype. Pediatr Endocrinol Rev.

[27] Li W (2017). Study of two Chinese families affected with resistant ovarian syndrome resulted from novel mutations of FSHR gene. Zhonghua yi xue yi chuan xue za zhi = Zhonghua yixue yichuanxue zazhi = Chinese journal of medical genetics.

[28] Wunsch A, Sonntag B, Simoni M (2007). Polymorphism of the FSH receptor and ovarian response to FSH. Annales D Endocrinologie.

[29] Perez Mayorga M (2000). Ovarian response to follicle-stimulating hormone (FSH) stimulation depends on the FSH receptor genotype. J Clin Endocrinol Metab.

[30] Greb RR (2005). A common single nucleotide polymorphism in exon 10 of the human follicle stimulating hormone receptor is a major determinant of length and hormonal dynamics of the menstrual cycle. J Clin Endocrinol Metab.

[31] Pabalan N (2014). Evaluating influence of the genotypes in the follicle-stimulating hormone receptor (FSHR) Ser680Asn (rs6166) polymorphism on poor and hyper-responders to ovarian stimulation: a meta-analysis. J Ovarian Res.

[32] Dragojevic-Dikic S (2010). An immunological insight into premature ovarian failure (POF). Autoimmun Rev.

[33] Chiauzzi VA (2004). Circulating immunoglobulins that inhibit the binding of follicle-stimulating hormone to its receptor: a putative diagnostic role in resistant ovary syndrome?. Clin Endocrinol.

[34] Meldrum DR (1980). Ovarian and adrenal steroidogenesis in a virilized patient with gonadotropin-resistant ovaries and hilus cell hyperplasia. Obstet Gynecol.

[35] Blecher M (1984). Receptors, antibodies, and disease. Clin Chem.

[36] Escobar ME (1982). Development of the gonadotrophic resistant ovary syndrome in myasthenia gravis: suggestion of similar autoimmune mechanisms. Acta Endocrinol.

[37] Chiauzzi V (1982). Inhibition of follicle-stimulating hormone receptor binding by circulating immunoglobulins. J Clin Endocrinol Metab.

[38] Riestenberg C, Ahern S, Shamonki M (2020). Follicle-stimulating hormone receptor autoantibody associated primary ovarian insufficiency successfully treated with corticosteroids: a case report. F S Rep.

